# Construction of a pharmaceutical care mode for cancer pain patients in primary care based on the Delphi method: an effective analysis

**DOI:** 10.3389/fphar.2023.1268793

**Published:** 2023-11-21

**Authors:** Han Xie, Xinyi Chen, Min Xue, Huaying Li, Yonghan Ge, Weihong Ge

**Affiliations:** ^1^ Department of Pharmacy, Nanjing Drum Tower Hospital, Nanjing, Jiangsu, China; ^2^ Department of Pharmacy, Nanjing Drum Tower Hospital Clinical College of Nanjing Medical University, Nanjing, Jiangsu, China; ^3^ Department of Pharmacy, Nanjing Yuhuatai District Xishanqiao Community Health Service Center, Nanjing, Jiangsu, China

**Keywords:** cancer pain, pharmaceutical care, Delphi method, pain management, primary care

## Abstract

**Objective**: Pain is one of the most common symptoms of cancer patients. Patients with advanced stages of cancer are always transferred to primary medical institutions or treated with home medication due to their specific pathophysiological characteristics. Studies have shown that continuous pharmaceutical care can improve the effectiveness and safety of drug therapy for cancer pain patients in primary care, but no relevant research has been conducted in China. Based on the Delphi method, this study aims to construct a pharmaceutical care mode for cancer pain patients and analyze its effect in drug therapy treatment in primary care in China.

**Methods:** A pharmaceutical care mode for cancer pain patients in primary care was developed through two rounds of expert consensus. A total of 200 cancer pain patients from January 2022 to January 2023 in Nanjing Drum Tower Hospital were recruited and divided into an intervention group and control group. The self-developed pharmaceutical care mode in primary care was conducted in the intervention group, while the traditional pharmaceutical care mode was conducted in the control group. Comparisons between the groups were performed in terms of pain assessment rate, reasonable rate of pain assessment, pain score, and incidence of adverse reactions.

**Results:** The initiative of experts in the two rounds of consultation was 100%, with an authority coefficient of 0.83. The coordination coefficient of the second round was higher than that of the first round, indicating that the consistency of expert opinions was enhanced. There were 100 cases in each group, and 12 and 8 were lost to follow-up in the intervention group and control group, respectively. Compared with the control group, the intervention group had a significantly higher pain assessment rate, a reasonable rate of pain assessment, and a significantly lower pain score and incidence of adverse reactions.

**Conclusion:** Under the scientific and reasonable mode of pharmaceutical care for cancer pain patients at the primary level, standardized drug therapy could significantly enhance the efficacy of treatment, thereby improving the quality of life of patients.

## 1 Introduction

Malignant tumor remains a crucial and life-threatening public health issue in the 21st century. The latest global cancer statistics reveals that China accounts for 24% and 30% of new cases and fatal cases of cancer all over the world, respectively (Cao et al., 2021; Sung et al., 2021). Pain is one of the most common symptoms of cancer patients, with an incidence of about 25% in newly diagnosed cases and 60%–80% in advanced cases. In addition, 1/3 patients with advanced stages of cancer suffer from moderate–severe pain (Chapman et al., 2020). Because of the characteristics of long-lasting effects and greater difficulty in treatment, cancer pain will significantly amplify both physical and psychological pain and reduce the quality of life of cancer patients without effective and timely control (Swarm et al., 2019; Zhang et al., 2021). The mean values of patients who report inadequate analgesics or dissatisfaction with pain management are 39.1 % and 40.3 % in North America and Europe, respectively (Liu et al., 2020). However, between 41.3% and 52.9% of patients report this situation in China (Liu et al., 2020). At present, drug therapy is the leading way to control cancer pain. Studies have proven that standardized medication treatment can alleviate 80%–90% of pain and improve the effect of analgesic treatment among cancer pain patients (Norman et al., 2017; Kawaguchi et al., 2018).

It is necessary for patients with cancer pain to receive quality pharmaceutical care to control the pain. However, malignant tumor progression indicates that most cancer pain patients are no longer suitable for active anti-cancer treatment in third-grade class-A hospitals but should be transferred to primary medical institutions or take medication at home. Due to the lack of a standardized pharmaceutical care mode and pharmaceutical care capacity, there appear many drug-related problems (DRPs) in the treatment of cancer pain in primary healthcare institutions in China. In addition, there remain insufficient effective connections between superior hospitals and primary medical institutions, resulting in a higher risk of medication among patients after referral, thereby restricting the effect of cancer pain management at the primary care and increasing the incidence of inadequate analgesia or adverse reactions. Masami et al. (Yamada et al., 2018) provided continuous cancer pain pharmaceutical care and confirmed that it could effectively reduce the degree of pain and the incidence of adverse drug reactions.

Based on the abovementioned analysis, this study first constructed a pharmaceutical care mode for cancer pain patients at the primary care level in China based on the Delphi expert consensus in order to focus on the continuity and quality of pharmaceutical care after referral from superior medical institutions to primary healthcare institutions. Second, this study carried out a prospective interventional study to investigate the effect of such modes so as to provide reference for promoting standardized and scientific practice of cancer pain pharmaceutical care at the primary care level.

## 2 Methods

### 2.1 Implementation of the Delphi procedure

#### 2.1.1 Formation of a research team

To ensure professionalism, the research team consisted of four members, including three senior clinical pharmacists and one master majoring in pharmacy. The main tasks included the preliminary construction of a pharmaceutical care mode for cancer pain patients at the primary care level, development of expert consultation questionnaires, determination of inquiry experts, distribution and collection of questionnaires, and statistical analysis.

#### 2.1.2 Composition of the experts

In this study, we recruited people from different positions in the field of cancer pain management, including clinical, pharmaceutical, and nursing experts, hospital administrators, pharmaceutical administration, health management research experts, and officials of health administration departments. The inclusion criteria were as follows: 1) more than 3 years of work experience in the field of cancer pain management, 2) bachelor’s degree and above, 3) medium-grade professional title and above, 4) and the ability to cooperate and complete two rounds of expert consultation.

#### 2.1.3 Expert consultation questionnaires

We searched related articles on continuous pharmaceutical care, cancer pain pharmaceutical care, and primary pharmaceutical care in the past 10 years on PubMed, Embase, Web of Science, China National Knowledge Infrastructure (CNKI), and Wangfang databases. On the basis of practical work experience, the research team summarized the key elements of the working mode and preliminarily set up a consultation questionnaire, including three first-level items and 16 second-level items. The questionnaire was composed of four parts: instructions, basic information of experts, construction index of the pharmaceutical care mode for cancer pain patients at the primary care level, and judgment basis and familiarity of experts. The Likert 5-level scoring method was used to determine the importance of indicators, and an open-ended supplementary suggestion column was set under each item, where experts could modify, supplement, or delete the corresponding items.

#### 2.1.4 Distribution and collection of questionnaires

Questionnaires were distributed by the “Wenjuanxing” platform and were collected within five working days for each round of consultation. The initiative of experts was expressed as the response rate of the questionnaires. We used the authority coefficient (Cr) to quantify the authority level among experts in the consultation, and the Cr was calculated as the average of familiarity with the questionnaire (Cs) and judgment coefficient (Ca). Kendall’s coefficient of concordance was used to evaluate the degree of coordination among experts, and the mean and standard deviation (SD) of each indicator reflected the concentration degree of experts’ opinions. Indicators were screened with the standard of the mean of importance assignment ≥3.5 and the coefficient of variation ≤0.3. Combining with supplementing and deleting the indicators according to the textual opinions of experts, the second round of consultation was formed. Based on the results of two rounds of consultation, a set of pharmaceutical care modes for cancer pain patients at the primary care level was obtained.

### 2.2 Empirical study on the primary cancer pain pharmaceutical care mode based on the Delphi method

#### 2.2.1 Study population

The institutional review board of Nanjing Drum Tower Hospital approved this study (No. 2022-664-02), and all participants signed informed consent. In our study, an effect size of 0.5 and a power of 0.9 were applied (Cohen, 1988; Dell et al., 2002). Considering the rate of loss to follow-up and the actual volume of cancer pain patients in our hospital, we decided to enroll 100 patients in each group. Then, a total of 200 cancer pain patients from January 2022 to January 2023 in our hospital were recruited for a prospective interventional study. The inclusion criteria were as follows: 1) confirmed diagnosis of cancer pain, 2) voluntarily participated and signed the informed consent, 3) expected survival time of more than 6 months, and 4) required continued cancer pain management after discharge. Patients were excluded if 1) they had cognitive dysfunction and could not communicate normally, 2) they were under 18 years of age, or 3) they could not cooperate with the regular pain follow-up.

Patients were divided into an intervention group (*n* = 100) and control group (*n* = 100). A study on the control group was carried out first, followed by the intervention group after some preparations, such as standardized cancer pain pharmaceutical care training.

#### 2.2.2 Empirical research methods

**FIGURE 1:**
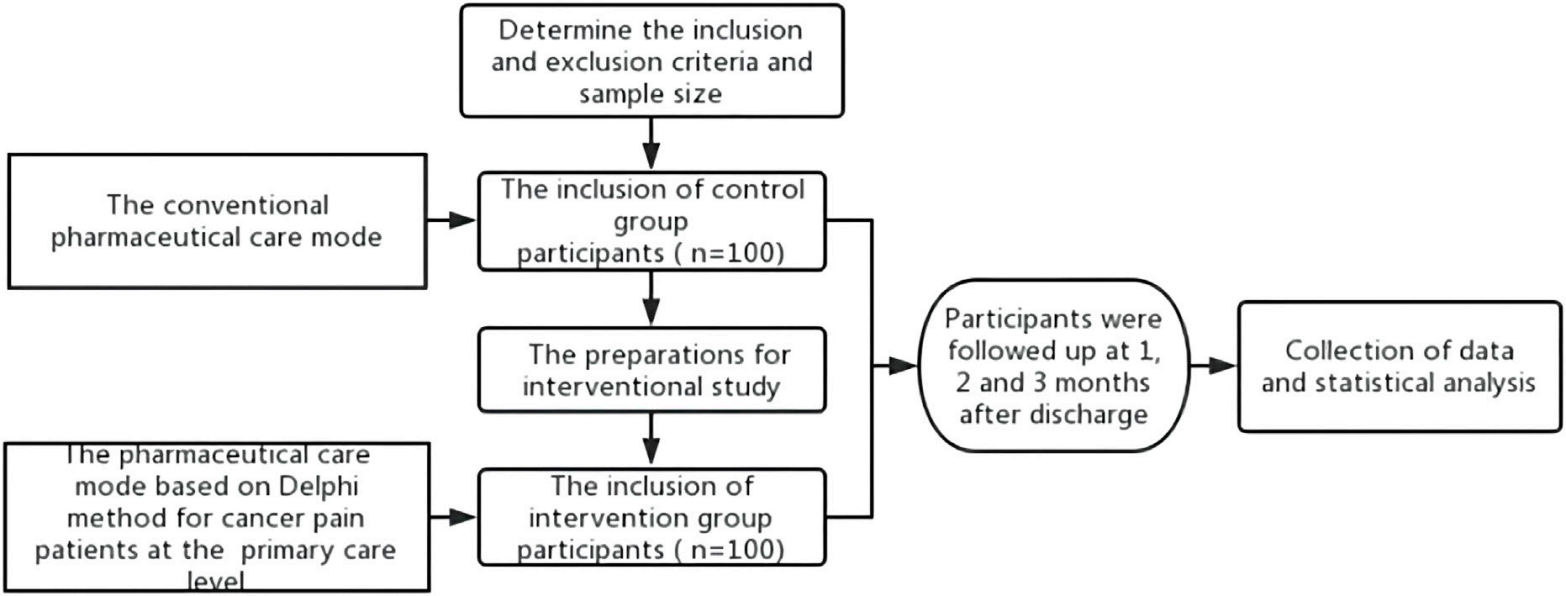
Flow chart of the empirical study.

**FIGURE 2:**
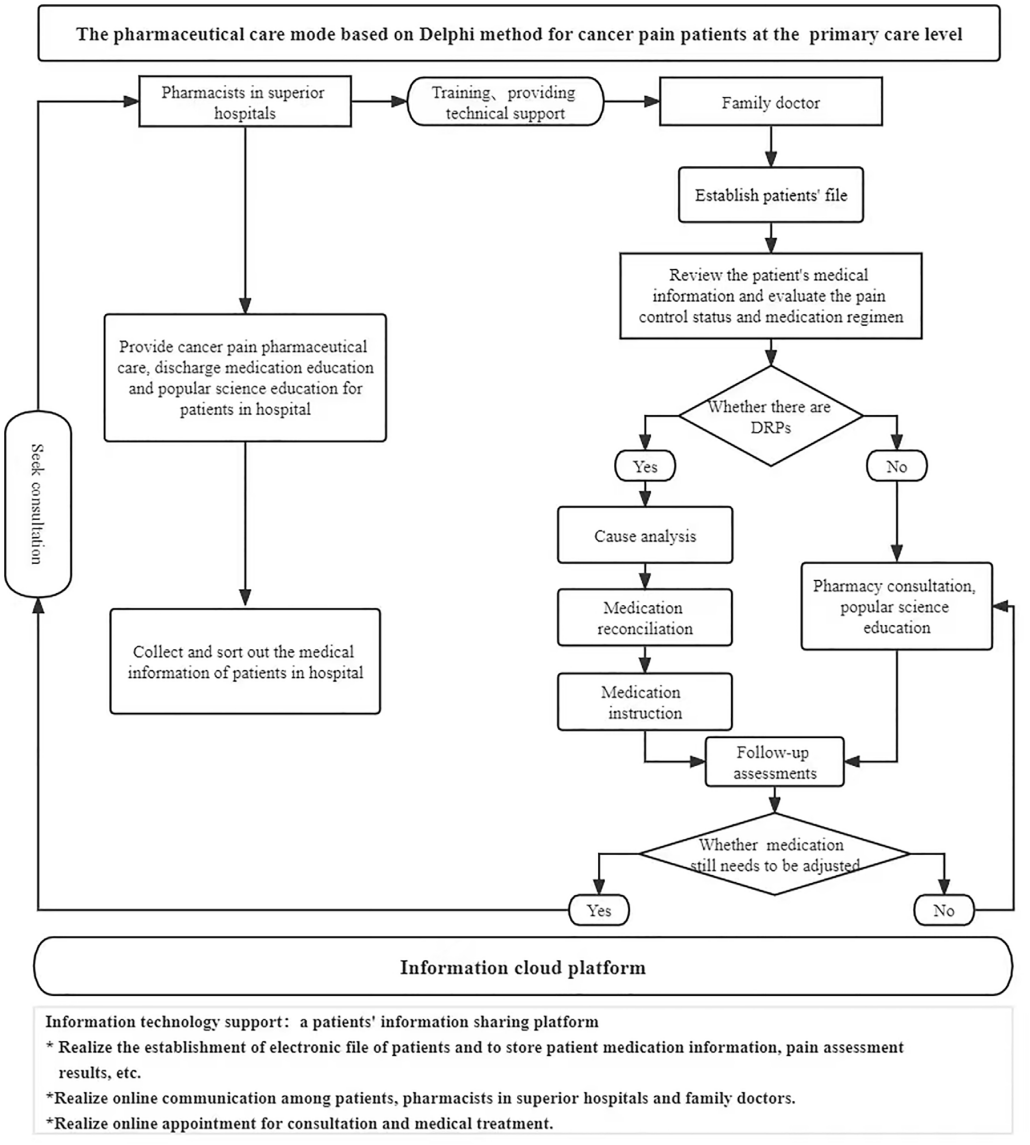
Pharmaceutical care mode for cancer pain patients at the primary care level in the intervention group.

In the control group, the conventional pharmaceutical care for cancer pain was conducted in addition to the extra telephone follow-up, i.e., there is no intervention in the existing pharmaceutical care mode, and both superior pharmacists and family doctors provide pharmaceutical care for the patients. Before the study on the intervention group, according to the pharmaceutical care mode formulated by the Delphi method, pharmacists in superior hospitals carried out training on standardized pharmaceutical care for cancer pain for family doctors in cooperative primary medical institutions and formulated relevant assessment contents and requirements. Only after family doctors completed the training and obtained the qualification certification, they could provide pharmaceutical care for cancer pain patients at the primary care level in the intervention group. Pharmacists in superior hospitals handed over the management of discharged patients to family doctors in the primary institutions and linked up the process of pharmaceutical care. When patients were in superior hospitals, pharmacists in superior hospitals provided cancer pain management, medication education at discharge, and popular science education. Also, they collected and sorted out the patient’s diagnosis and treatment information in time and uploaded it to the information cloud platform for family doctors to check after contacting the patients. After patients were referred to primary medical institutions or received home drug treatment, the family doctor established a file for them and evaluated the status of pain control and existing medication regimens. Family doctors monitored the DRPs in the process of drug treatment for cancer pain and provided patients with medication reconciliation, follow-up assessment, medication guidance, pharmaceutical consultation, and popular science education. Patients’ medication information, pain score, and other information can be uploaded by family doctors or patients independently to the information cloud platform for sharing. When encountering difficult cases, family doctors can seek consultation from superior pharmacists through the information cloud platform to jointly manage the patient’s drug treatment process. Additional working details are reflected in the provided figures. The flow chart of the empirical study is shown in Figure 1, and the pharmaceutical care mode conducted in the intervention group is displayed in Figure 2.

#### 2.2.3 Observation indicators

Comparisons between the intervention group and control group after different pharmaceutical care modes were performed using the following indicators: 1) pain score: numerical rating scale (NRS) was used to evaluate the pain status after pharmaceutical care for 1 month, with 0 point indicating painless, 1–3 points indicating mild pain, 4–6 points indicating moderate pain, and 7–10 points indicating severe pain (Fink and Gallagher, 2019); 2) pain assessment rate: pain assessment during analgesic drug treatment. Pain assessment rate = (number of patients with pain assessment/total number of patients in the group) * 100%; 3) reasonable rate of pain assessment: reasonable pain assessment was not only the assessment of pain intensity but also the comprehensive assessment of pain site, pain nature, and previous medication regimen (Magee et al., 2019). The pharmacist of the superior hospital would judge the pain assessment. Reasonable rate of pain assessment = (number of reasonable pain assessments/total number of pain assessments) * 100%; and 4) incidence of adverse reactions: adverse reactions during analgesic drug treatment, including constipation, nausea, vomiting, dizziness, pruritus, and urinary retention. (Gahr et al., 2017). Incidence of adverse reactions = (number of patients with adverse reactions/total number of patients in the group) * 100%.

### 2.3 Statistical analysis

Variables were expressed as mean ± standard deviation (x ± s) and counts (proportion) for continuous and categorical variables separately. Numerical differences between the two groups were assessed by the chi-squared test for categorical variables and the *t*-test for continuous variables. The threshold for significance was *p* = 0.05. Excel 2021 software was used for data input. All statistical analyses were conducted using SPSS version 25.0 (SPSS Inc., Chicago, IL, USA).

## 3 Results

### 3.1 Results of Delphi

There were 24 experts in each round of expert consultation, and their basic information is shown in Table 1. Related fields included clinical pharmacy, pharmacy administration, hospital pharmacy, general medicine, geriatrics, nursing, clinical pharmacy, big data, and other fields.

**TABLE 1:** Experts’ basic information (*n* = 24).

Basic information	Classification	Number	Percentage (%)
Gender	Male	14	58.33
	Female	10	41.67
Highest academic qualification	Doctor	8	33.33
	Master	6	25
	Bachelor	10	41.67
Title	Senior	8	33.33
	Deputy senior	6	25
	Intermediate	10	41.67
Years of work in the field of cancer pain management	3–9	6	25
	10–16	6	25
	17–23	6	25
	24–30	6	25

The response rate of valid questionnaires represented the initiative of experts. We collected 24 valid questionnaires in each round, and the response rate was 100.0%, with an authority coefficient of 0.83. Kendall’s coefficient of concordance of the two rounds was 0.237 and 0.444 (*p* < 0.01), indicating a statistically significant difference (see Table 2).

**TABLE 2:** Kendall’s coefficient of concordance of the two rounds.

	χ^2^	*p*	Kendall’s coefficient of concordance (W)
Round 1	99.516	0.000^*^	0.237
Round 2	37.276	0.000^*^	0.444

*P* < 0.05 indicates a statistically significant difference.

We collected experts’ evaluation of each item and their opinions on literal adjustment in the two rounds of questionnaires, excluding indicators with arithmetic mean <3.5 and coefficient of variation >0.3. Finally, three primary indicators and 14 secondary indicators were selected (see Appendix 1). Based on this indicator system, we preliminarily constructed a pharmaceutical care mode for cancer pain patients at the primary care level and applied it to the intervention group in the empirical study (see Figure 2).

### 3.2 Results of empirical research

#### 3.2.1 General information

A total of 200 cancer pain patients were recruited in this study, with 100 cases in the intervention group and 100 cases in the control group. Due to migration, death, or voluntary withdrawal, 20 patients were lost to follow-up. Finally, there were 88 patients in the intervention group and 92 in the control group. In terms of tumor types, most patients had pancreatic cancer, gastric cancer, intestinal cancer, lung cancer, liver cancer, and biliary tract cancer. Very few patients had breast cancer and throat cancer, which were considered as other cancer types. No significant differences were found between the two groups in gender, age, body mass index (BMI), tumor type, and other general information, with all *p*-values greater than 0.05. See Table 3 for details.

**TABLE 3:** General information of patients in both groups.

Clinical parameter		Control group	Intervention group (*n* = 88)	p
		(*n* = 92)		
Gender				0.267^^^
	Male	54.3% (50/92)	62.5% (55/88)	
	Female	45.7% (42/92)	37.5% (33/88)	
Age		58.3 ± 10.5	57.2 ± 9.0	0.458^^^
BMI (kg/m^2^)		20.6 ± 3.4	21.8 ± 3.8	0.061^^^
Tumor types				
	Pancreatic caner	27.2% (25/92)	25.0% (22/88)	0.740^^^
	Intestinal cancer	12.0% (11/92)	10.2% (9/88)	0.712^^^
	Lung cancer	10.9% (10/92)	10.2% (9/88)	0.889^^^
	Gastric cancer	23.9% (22/92)	20.5% (18/88)	0.577^^^
	Liver cancer	7.6% (7/92)	10.2% (9/88)	0.537^^^
	Biliary system cancer	13.0% (12/92)	17.0% (15/88)	0.452^^^
	Other	5.4% (5/92)	6.8% (6/88)	0.699^^^

*P* > 0.05 indicates no statistically significant difference.

#### 3.2.2 Observation indicators

##### 3.2.2.1 Pain assessment rate and reasonable rate of pain assessment

Pain assessment was conducted after 1-month implementation of the cancer pain pharmaceutical care mode. As shown in Table 4, the intervention group had a significantly higher pain assessment rate and a reasonable rate of pain assessment compared to the control group.

**TABLE 4:** Pain assessment rate and reasonable rate of pain assessment in both groups.

Group	Pain assessment rate		Reasonable rate of pain assessment	
	Assess	Not assess	Reasonable assessment	Unreasonable assessment
Control group (n = 92)	58.7% (54/92)	41.3% (38/92)	74.1% (40/54)	25.9% (14/54)
Intervention group (n = 88)	98.9% (87/88)	1.1% (1/88)	97.7% (85/87)	2.3% (2/87)
χ^2^	42.758		18.489	
*p*	0.000^*^		0.000^*^	

*P* < 0.05 indicates a statistically significant difference.

##### 3.2.2.2 Pain score

Pain scores were collected at 1–3 months after the implementation of the cancer pain pharmaceutical care mode. As shown in Table 5, no significant difference between the two groups was found in the 1-month follow-up. However, at 2-month and 3-month follow-up analyses, the intervention group had a significantly lower pain score compared to the control group.

**TABLE 5:** Comparison of pain scores between the two groups.

Group	NRS (1-month)	NRS (2-month)	NRS (3-month)
Control group (n = 92)	3.10 ± 1.57	3.08 ± 1.75	2.97 ± 1.66
Intervention group (n = 88)	3.02 ± 1.59	2.59 ± 1.45	2.44 ± 1.36
t	0.319	2.020	2.315
*p*	0.750	0.045^*^	0.022^*^

*P* < 0.05 indicates a statistically significant difference.

##### 3.2.2.3 Incidence of adverse reactions

Adverse reactions were recorded after 3 months of the implementation of the cancer pain pharmaceutical care mode. As shown in Table 6, compared with the control group, the intervention group had a significantly lower incidence of adverse reactions.

**TABLE 6:** Comparison of the incidence of adverse reactions between the two groups.

Group	Constipation	Nausea	Vomiting	Vertigo	Pruritus	Urinary retention	Total incidence
Control group (*n* = 92)	26.1% (24/92)	19.6% (18/92)	10.9% (10/92)	6.5% (6/92)	1.1% (1/92)	1.1% (1/92)	65.2% (60/92)
Intervention group (*n* = 88)	19.3% (17/88)	10.2% (9/88)	3.4% (3/88)	3.4% (3/88)	2.3% (2/88)	0.0% (0/88)	38.6%^*^ (34/88)

*P* < 0.05 indicates a statistically significant difference.

## 4 Discussion

### 4.1 Scientific nature of the pharmaceutical care mode for cancer pain patients in primary care based on the Delphi method

As a mature and reliable method that has been widely applied in the field of health services, the Delphi method is often used in the formulation of models and standards (Boulkedid et al., 2011). On the basis of the Delphi method, Katie et al. (Earle-Payne et al., 2022) identified 44 practice standards for general clinical pharmacists to provide multiple medications and chronic disease drug treatment reviews and proved that such standards were acceptable and effective. Therefore, in this study, we used the Delphi method to construct a set of pharmaceutical care modes that could be suitable for cancer pain patients at the primary healthcare level in China.

It is of great importance for the Delphi procedure to select experts with higher attainments based on strict inclusion criteria (Shawahna, 2020). In this study, we recruited people from different positions in the field of cancer pain management to ensure good disciplinary representativeness, including clinical, pharmaceutical, and nursing experts, hospital administrators, pharmaceutical administration, health management research experts, and officials of health administration departments. In addition, 75% of the experts have worked in the field of cancer pain management for more than 10 years, which guarantees the professionalism and scientific nature of the research.

The response rates of the questionnaire in both rounds were 100%. In addition, the experts put forward a number of constructive suggestions, reflecting the high initiative and support of experts for this study (Diamond et al., 2014). The results remained the same in the two rounds of consultation, with an authority coefficient of 0.83 (i.e., greater than the cut-off value of 0.7), pointing out a higher degree of authority (Wang et al., 2019). Compared with the first round (0.237) of expert consultation, Kendall’s coefficient of concordance in the second round (0.444) was significantly higher, suggesting that after the first round of consultation and correction, the coordination degree of experts’ opinions in the second round of consultation increased, and the consistency was better. After two rounds of consultation, three primary indicators and 14 secondary indicators were selected by combining the screening criteria, expert opinions, and statistical results. A set of pharmaceutical care paths for cancer pain patients at the primary care level with unified standards and complementary advantages was formed.

### 4.2 Standardizing the pain assessment among cancer pain patients at the primary care level

Pain assessment is the first step in the management of cancer pain. Only by making a reasonable dynamic pain assessment and understanding the characteristics of pain degree, site, nature, and psychological state can the corresponding medication regimen be formulated so as to improve the treatment effect of cancer pain and the quality of life of patients. Sun et al. (2018) conducted an intervention–control study with either standardized or conventional pain assessment in the two groups, and the results showed that compared with the control group, the intervention group had a significantly lower pain score, depression score, and anxiety score but higher quality of life and satisfaction.

Most pain assessment in the community among cancer pain patients in developed countries is completed by pharmacists (Savage et al., 2013). At present, China is advocating and promoting pharmacists to join the family doctor team to manage primary healthcare through multidisciplinary collaboration, which also requires improvement of the standardization of pain assessment by the whole family doctor team. The mode constructed in this study emphasized the training and guidance of pharmacists from superior medical institutions to family doctors, among which pain assessment was an important part of standardized cancer pain management. Family doctors in our study were all trained before providing standardized cancer pain pharmaceutical care for the intervention group, and the results revealed that the intervention group had a significantly higher pain assessment rate and reasonable rate of pain assessment than the control group, which laid the foundation for the implementation of accurate, timely, and effective drug treatment.

### 4.3 Effectiveness and safety of cancer pain management by the pharmaceutical care mode for cancer pain at the primary care level based on the Delphi method

Pharmaceutical care in the management of cancer pain medication abroad includes the assessment of pain control, number of acute pain attacks, adverse drug reactions, formulation of medication regimens, and management of medication adherence (Burns and Anne, 2008). Some studies have also pointed out that the main reason for the poor pain control in China might be the irrational use of analgesic drugs, misunderstanding of analgesic drugs by patients and their families, difficulty in obtaining pain management services, and poor adherence with analgesic drugs (Xu et al., 2018). In view of the abovementioned problems and referring to cancer pain medication management in other countries, our pharmaceutical care mode standardized the process of primary pharmaceutical care, promoted collaboration between superior and primary health institutions, and provided pharmaceutical care for patients, such as pain assessment, medication management, medication education, and public education.

Pain scores were analyzed at 1–3 months after the implementation of the cancer pain pharmaceutical care mode. No significant difference between the intervention and control groups was found in the 1-month follow-up, which might be related to the low degree of cooperation and trust between patients and family doctors. However, at 2-month and 3-month follow-up, the intervention group had a significantly lower pain score compared to the control group, proving that the self-developed mode could improve the effectiveness of cancer pain control. The majority of patients prefer to visit hospitals because of their concerns about the service level of primary medical care. Under the standardized pharmaceutical care mode, family doctors gradually penetrate into the process of patient diagnosis and treatment, so patients can experience the same service from primary care as hospitals and will be more willing to seek cancer pain management from family doctors. The intervention group in our study had a significantly lower incidence of adverse reactions compared to the control group after 3 months of the implementation of the cancer pain pharmaceutical care mode, noting that such modes could remarkably increase the safety of cancer pain drug treatment. Standardizing the path of pharmaceutical care and clarifying the responsibilities and workflow of family doctors is conducive to timely detection of drug-related events and adjustment of medication regimens, thus conducting individualized management of cancer pain.

Medication adherence remains an influencing factor for the effect of cancer pain medication. In our study, both the intervention and control groups had more than half of patients with moderate adherence, without statistical difference. However, compared with the control group, the intervention group had significantly more patients with good adherence and less with poor adherence. The abovestated results demonstrated that through standardized pharmaceutical care for cancer pain at the primary care level, regular medication evaluation records and medication education for patients could improve their medication adherence.

### 4.4 Limitations

There are some limitations in this study. First, 20 of the 24 experts in consultation were from Nanjing, Jiangsu Province, which had regional characteristics. Thus, the pharmaceutical care mode constructed in this study may need to be adjusted and improved according to the actual situation in the future. Second, empirical research was a single-center study with a relatively small sample size and short follow-up time, which might lead to bias. We expect to expand the research scope and sample size in the process of promotion and application in the later stage and to add the research on the influence of the application of this mode on patient medication adherence and economics so as to further confirm the application effect of this mode in cancer pain management in the primary care level.

## 5 Conclusion

Through two rounds of Delphi expert consensus, this study developed a set of pharmaceutical care modes for cancer pain at the primary care level, which standardized the management process of cancer pain medication for patients referred to primary medical institutions or at home. The empirical research results of this model indicated that our pharmaceutical care mode could reduce the risk of drug use in cancer pain patients, as well as improve the quality of cancer pain management at the primary care level. We recommend further promoting and applying this mode in clinical practice in order to achieve continuous improvement and optimization.

## Data Availability

The raw data supporting the conclusion of this article will be made available by the authors, without undue reservation.

## Appendix 1 Indicator system of conducting the pharmaceutical care mode for cancer pain patients in primary care

**Table:**

Primary indicators	Secondary indicators
A. Pharmacists in superior hospitals	A1. Provide cancer pain pharmaceutical services, discharge medication education, and popular science education for patients
	A2. Sort out the diagnosis–treatment information and medication history of patients in the hospital and upload it in time; establish the electronic file of patients (realize the sharing of patients’ diagnosis and treatment information with family doctors)
	A3. Provide training on cancer pain drug treatment management for primary family doctors and organize qualification certification (guard the level of cancer pain pharmaceutical service in primary care)
	A4. Provide technical support to primary family doctors and participate in difficult case consultations initiated by family doctors
B. Family doctor	B1. Review the patient’s medical information and evaluate the pain control status and medication regimen
	B2. Analyze the causes of DRPs, carry out medication reorganization and other work, and give medication instruction to patients
	B3. Provide pharmaceutical consultation and popular science education for patients
	B4. Follow-up regularly to evaluate the effect of pain management and to determine whether the current analgesic regimen needed to be adjusted
	B5. Record and upload patients’ medication information and assessment results regularly
	B6. Improve their pharmaceutical service ability and participate in relevant training and examination actively
	B7. Strengthen cooperation and communication with doctors, pharmacists, and other medical personnel in relevant departments of superior hospitals
C. Information technology support	C1. Build a patients’ information sharing platform to realize the establishment of electronic files of patients and to store patient medication information, pain assessment results, etc.
	C2. Realize online communication among patients, pharmacists in superior hospitals, and family doctors
	C3. Realize online appointments for consultation and medical treatment
